# Surveillance strategies for the detection of new pathogen variants across epidemiological contexts

**DOI:** 10.1371/journal.pcbi.1012416

**Published:** 2024-09-05

**Authors:** Kirstin I. Oliveira Roster, Stephen M. Kissler, Enoma Omoregie, Jade C. Wang, Helly Amin, Steve Di Lonardo, Scott Hughes, Yonatan H. Grad

**Affiliations:** 1 Department of Immunology and Infectious Diseases, Harvard T.H. Chan School of Public Health, Boston, Massachusetts, United States of America; 2 Center for Communicable Disease Dynamics, Harvard T.H. Chan School of Public Health, Boston Massachusetts, United States of America; 3 Department of Computer Science, University of Colorado Boulder, Boulder, Colorado, United States of America; 4 New York City Department of Health and Mental Hygiene, New York City, New York, United States of America; CPERI, GREECE

## Abstract

Surveillance systems that monitor pathogen genome sequences are critical for rapidly detecting the introduction and emergence of pathogen variants. To evaluate how interactions between surveillance capacity, variant properties, and the epidemiological context influence the timeliness of pathogen variant detection, we developed a geographically explicit stochastic compartmental model to simulate the transmission of a novel SARS-CoV-2 variant in New York City. We measured the impact of (1) testing and sequencing volume, (2) geographic targeting of testing, (3) the timing and location of variant emergence, and (4) the relative variant transmissibility on detection speed and on the undetected disease burden. Improvements in detection times and reduction of undetected infections were driven primarily by increases in the number of sequenced samples. The relative transmissibility of the new variant and the epidemic context of variant emergence also influenced detection times, showing that individual surveillance strategies can result in a wide range of detection outcomes, depending on the underlying dynamics of the circulating variants. These findings help contextualize the design, interpretation, and trade-offs of genomic surveillance strategies of pandemic respiratory pathogens.

## Author summary

To prevent the spread of infections that are more transmissible, evade immunity, or cause more serious illness, public health agencies must quickly detect changes in pathogens such as the virus responsible for COVID-19, which is done by testing the population to identify infections and then sequencing the positive cases to determine which virus variants caused the infections. However, it is unclear how different factors, such as the volume of testing and sequencing, the timing in the outbreak, or the transmissibility of the new variants affect our ability to quickly detect new variants of concern. In our study, we used mathematical simulations of disease spread in New York City to better understand how these factors influence the time it takes to detect a new variant and how many people have been infected by the time it is detected. In our simulations, the greatest improvement in detection speed was achieved by increasing the number of positive cases that are sampled for sequencing. However, factors beyond policymakers’ control also influenced the time it took to detect the new variant, meaning that a wide range of detection outcomes was possible even under an ideal public health strategy. These findings help guide decision making for future outbreaks.

## Introduction

The COVID-19 pandemic highlighted the importance of genomic surveillance as a tool to detect and characterize novel genetic variants of pandemic pathogens, monitor their relative prevalence, and update diagnostics and vaccines [1–7]. Identifying new variants of concern (VOCs) as early as possible helps public health agencies update nonpharmaceutical countermeasures, therapeutics, and forecasts, and implement interventions to reduce the spread of infections that are potentially more transmissible or more immune evasive and that lead to more severe outcomes than prior variants. As the availability of sequencing technology expands, guidelines for sampling and variant detection—not only of SARS-CoV-2 but also other respiratory pathogens with pandemic potential—form an integral component of pandemic response.

Challenges in designing effective surveillance systems include determining appropriate sample sizes and ensuring that samples are representative of the pool of infections, which is complicated by geographic and temporal variation in case definitions, testing guidelines, and testing capacity. Sample representativeness may also be affected by the rate of asymptomatic infections, severity of symptoms, and other variant characteristics as well as population immunity and human behavior, which may change as new variants emerge and the epidemic evolves.

Existing research has addressed some of these questions. Early in the COVID-19 pandemic, the European Centre for Disease Prevention and Control (ECDC) provided sample size calculations based on sampling theory to guide the detection of new variants before they reach a pre-specified proportion of all infections [8]. Wohl and colleagues expanded these calculations to account for variant biology and logistical factors, such as testing rates by symptom status, sample quality, and test sensitivity. They showed that detection likelihood and speed are affected by these variant-specific biases in sampling probabilities and should be accounted for in surveillance system design and data interpretation [9]. In addition to sampling theory, simulations have been implemented to assess the impact of specific surveillance decisions on variant detection. Contreras and colleagues focused on resource allocation between ports-of-entry and the broader community, highlighting the importance of adaptive strategies [10]. Han and colleagues explored the effect of testing volume on variant detection in settings with non-random sampling from sentinel sites. Their findings underscore the importance of approximating population sampling and reaching sufficiently high test volume before expanding sequencing in low-resource settings [11]. Wegner and colleagues also measured the impact of sampling rates, using empirical genomic data from Switzerland. They found that the delay in variant detection at different levels of down-sampling was strongly lineage-dependent [12]. However, the combinations of sampling strategies, variants, and epidemiological settings that have been observed empirically in pandemic settings are limited, and many questions remain about the effects of surveillance decisions on variant detection.

Here, we expanded on this prior work and considered the role of geography, human mobility, epidemic stage, and sampling volumes, as well as their interactions. We developed a geographically explicit stochastic transmission model using empirical human mobility data to simulate the geographic dispersal of two SARS-CoV-2 variants across New York City (NYC). We chose COVID-19 in NYC as a case study given publicly available data on testing, sequencing, and mobility [13,14] and the City’s role in variant importation [15], but our model may be adapted to other locations and respiratory pathogens. We varied both the timing and location of introduction of the novel variant and its transmissibility relative to the preexisting variant. For each combination of surveillance strategy, epidemiological setting, and variant transmissibility, we measured the speed of new variant detection and the undetected disease burden. By developing this framework, we aimed to contextualize decision-making on genomic surveillance within the diversity of possible disease scenarios.

## Methods

### Data

Baseline COVID-19 testing rates (609 tests per 100,000 residents per week) and sequencing rates for NYC were obtained from the NYC Department of Health and Mental Hygiene (NYC DOHMH) [14] from December 2020 until November 2021 at the geographic resolution of modified ZIP-code tabulation areas (MODZCTAs). We obtained mobility data from Meta via the Facebook Data for Good Initiative [16], which reported the physical locations of anonymized app users within 600m-by-600m tiles in 8-hour intervals. We aggregated these data to boroughs and used them to construct a mixing matrix estimating the rate of interpersonal encounters among the residents of NYC. We defined mappings between MODZCTAs, tiles, and boroughs using United States Census Bureau data. Full details are provided in the **Supplementary Materials and Methods (S1 Text).**

### Transmission model

To simulate the transmission of a novel SARS-CoV-2 variant, we constructed a geographically explicit two-strain stochastic compartmental model. We used a stochastic model to account for randomness in transmission associated with the small initial number of infections with the novel strain. We assumed a closed population given the short time period considered in this study.

Individuals proceed through model states as follows (**S1 Fig**): Individuals are initially susceptible (*S*) and become exposed to one of two strains (*E*_1_, *E*_2_), upon contact with an infected individual (*I*_1_, *I*_2_). Contact may occur within and between locations, modeled as patches, at rates determined by empirically observed mobility patterns across NYC [16]. Each location represents a borough. Infections may remain undetected (*I*_*U*_), detected through testing (*I*_*T*_), or selected for sequencing after testing (*I*_*G*_). Sequencing a sample from an infection with the novel variant leads to variant detection, which is the main outcome of interest in this study. Individuals with a positive COVID-19 test (with or without a sequenced sample) choose to follow social distancing guidelines with a probability of *p*_*q*_, thus reducing their transmission probability to a proportion (*θ*) of the transmissibility of the base strain. Infections remain undetected if no COVID-19 test is reported or if the test produces a false negative result. Upon recovery, individuals are temporarily immune (*R*), before becoming susceptible to re-infection at a reduced rate reflecting cross-reactive immunity (*S*). A small portion of individuals isolate in response to a false positive test result (*S*_*q*_) and are removed from the pool of susceptible individuals. The novel SARS-CoV-2 strain has greater transmissibility, greater immune evasion, or both, but is otherwise assumed to be identical to the base strain in its incubation and recovery periods and detection probability. In sensitivity analyses, we considered varying contact rates (**S9 Fig**) and varying incubation periods of the novel variant (**S10 Fig**). Full details on the model structure and parameters are provided in the **Supplementary Materials and Methods (S1 Text)**. Code is available at github.com/gradlab/detecting-sarscov2-variants.

### Surveillance scenarios

We compared surveillance strategies that varied by the volume of testing and sequencing deployed, represented in the model as varying testing (*p*_*t*_) and sequencing probabilities, (*p*_*g*_). We considered a range of strategies for test distribution among locations, specifically (1) maintaining the way tests have been distributed historically in the data by NYC DOHMH (baseline test distribution), (2) distributing tests by population density, (3) randomly allocating tests among locations, and (4) over-sampling a single location (20–100% of tests) with the remainder of tests distributed among the remaining locations by population density. In a sensitivity analysis, we modeled fixed caps on sequencing capacity rather than sequencing proportions, to capture more realistically the resource constraints that may emerge during periods of high COVID-19 incidence. By definition, sequencing a fixed proportion of positive tests produces a greater number of sequenced samples when testing volume is increased, thus making it difficult to understand whether any improvements in variant detection are driven by testing (increased representativeness among the pool of positive tests) or the larger number of sequenced samples (and thus opportunities for selecting a sample from the strain of interest). This sensitivity analysis allowed us to vary testing volume without impacting the number of sequenced samples, helping to evaluate the contribution of testing *versus* sequencing to improvements in detection speed.

### Emergence scenarios

While testing and sequencing can be optimized, many factors affecting detection outcomes remain beyond the control of surveillance systems. In this model, we estimated to what extent the timing and location of variant emergence affected detection outcomes. Specifically, we varied the introduction time of the novel variant relative to the base variant, delaying introduction from 0 to 150 days. We also simulated introduction of the novel variant in all possible locations (boroughs) under each scenario of surveillance resource allocation, and compared situations where surveillance was targeted in the location where the novel strain emerged (surveillance scenario 4 described above) and assessed the importance of connectivity of the introduction location.

### Statistical analysis

The main outcomes in this study were the time to variant detection (the number of days between when the index case becomes infectious and laboratory confirmation of the new variant among sequenced specimens), the cumulative number of infections, and the variation in cumulative infections across locations. We ran 3,000 simulations per scenario—100 simulations for each combination of introduction time and location—and calculated the arithmetic means, medians, and confidence intervals of the main outcomes across simulations. We assessed whether distributions in detection outcomes were significantly different for different parameter values using a two-sided Wilcoxon rank-sum test. Finally, we compared the relative influence of surveillance strategy and emergence context variables, by conducting a multivariable linear regression of detection time on testing rates, sequencing rates, geographic allocation strategy, emergence location, emergence time, and transmission probability.

## Results

### Testing and sequencing volumes

**Fig 1:**
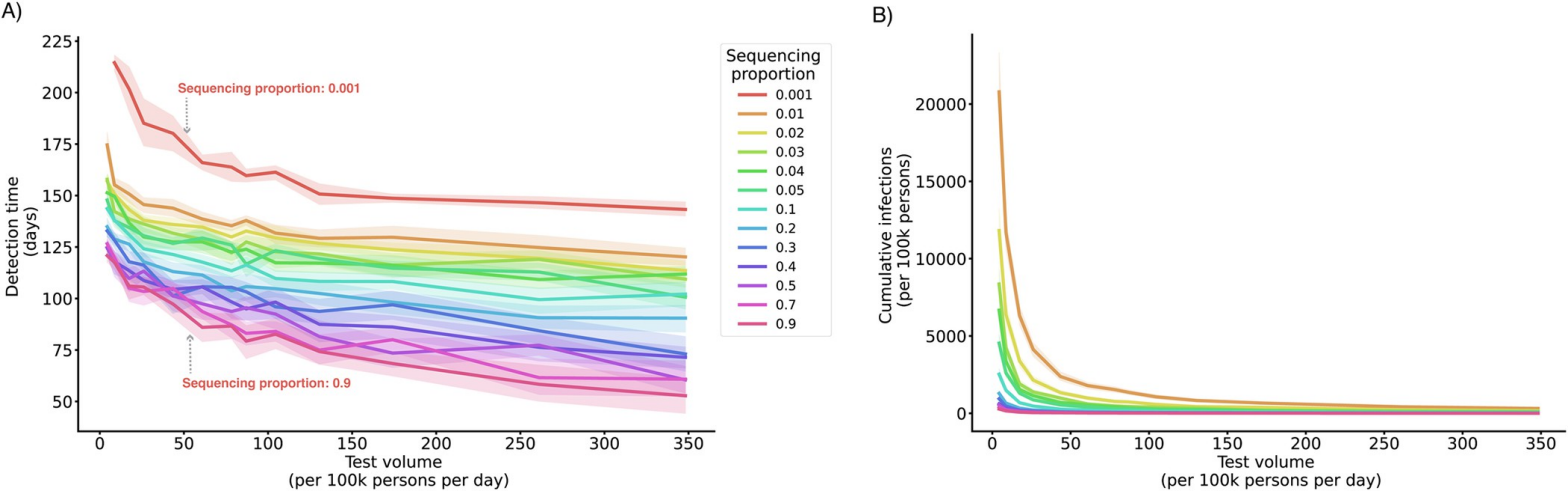
Detection outcomes by test quantity and sequencing rate. Lines depict the mean duration between variant introduction and detection in days (A) and the cumulative infections upon detection (B) as a function of daily testing volume (given new variant introduction 50 days after the prior variant, baseline test strategy). Shaded areas depict the 95% simulation interval for the detection time. Colors represent proportions of tests selected for sequencing.

Outcomes varied considerably across testing and sequencing rates. Higher rates led to faster detection, fewer cases, and less variation in cumulative infections across locations (**Fig 1**). In accordance with sampling guidelines for well-resourced settings [17], we assumed that a fixed percentage of tests was sequenced. Thus, by definition, increasing the number of tests also increased the number of sequenced samples.

**Fig 2:**
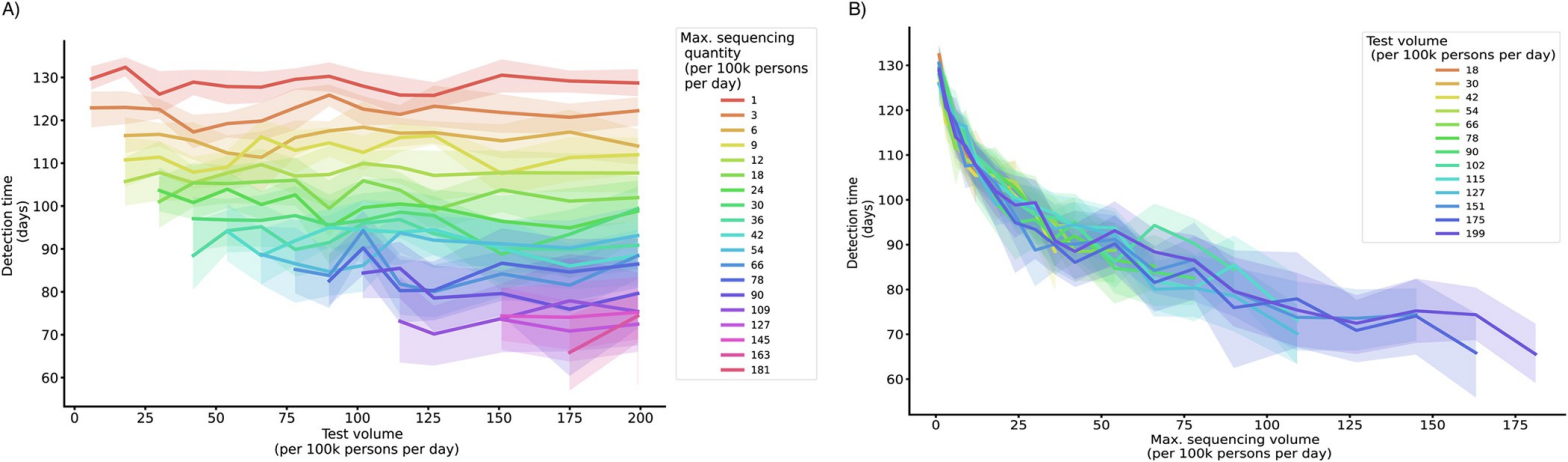
Detection time by test volume and fixed sequencing capacity. Lines depict the mean duration between variant introduction and detection in days as a function of daily testing volume, colored by the maximum sequencing volume (A), and as a function of daily maximum sequencing volume, colored by the test volume (B) (at variant introduction 50 days after the prior variant and baseline sampling strategy). Shaded areas depict the 95% simulation interval for the detection time.

To differentiate the individual contributions of testing and sequencing, we fixed the quantities of samples selected for sequencing at varying testing volumes. Fixed sequencing volumes were implemented as a cap on the maximum number of samples that can be sequenced per day, with the test positivity rate determining the number of sequenced cases up to the cap. At all levels of testing, increasing the number of sequenced samples reduced the detection time, while increasing testing alone had little impact on new variant detection speed (**Fig 2**). As such, the improvement in variant detection with increasing test volumes at a given sequencing proportion was driven by the increase in sequencing volume rather than test volume.

We also considered an alternative strategy for capping sequencing, in which the sequencing volume depended on both the test volume and the positivity rate (**S1 Text).** The results from this sensitivity analysis fall between the fixed volume and fixed rate analyses: raising testing capacity improved detection times for low levels of testing (up to 50–75 tests per 100k persons), whereas at higher levels of testing, improvements in detection time were driven primarily by increased sequencing capacity (**S4 Fig**). The first sensitivity analysis maximized variation in the effective sequencing rate to better compare the effect of raising testes *versus* sequencing, while the second sensitivity analysis represents a more realistic scenario of how a sequencing cap may be implemented in practice.

### Geographic sampling strategy

**Fig 3:**
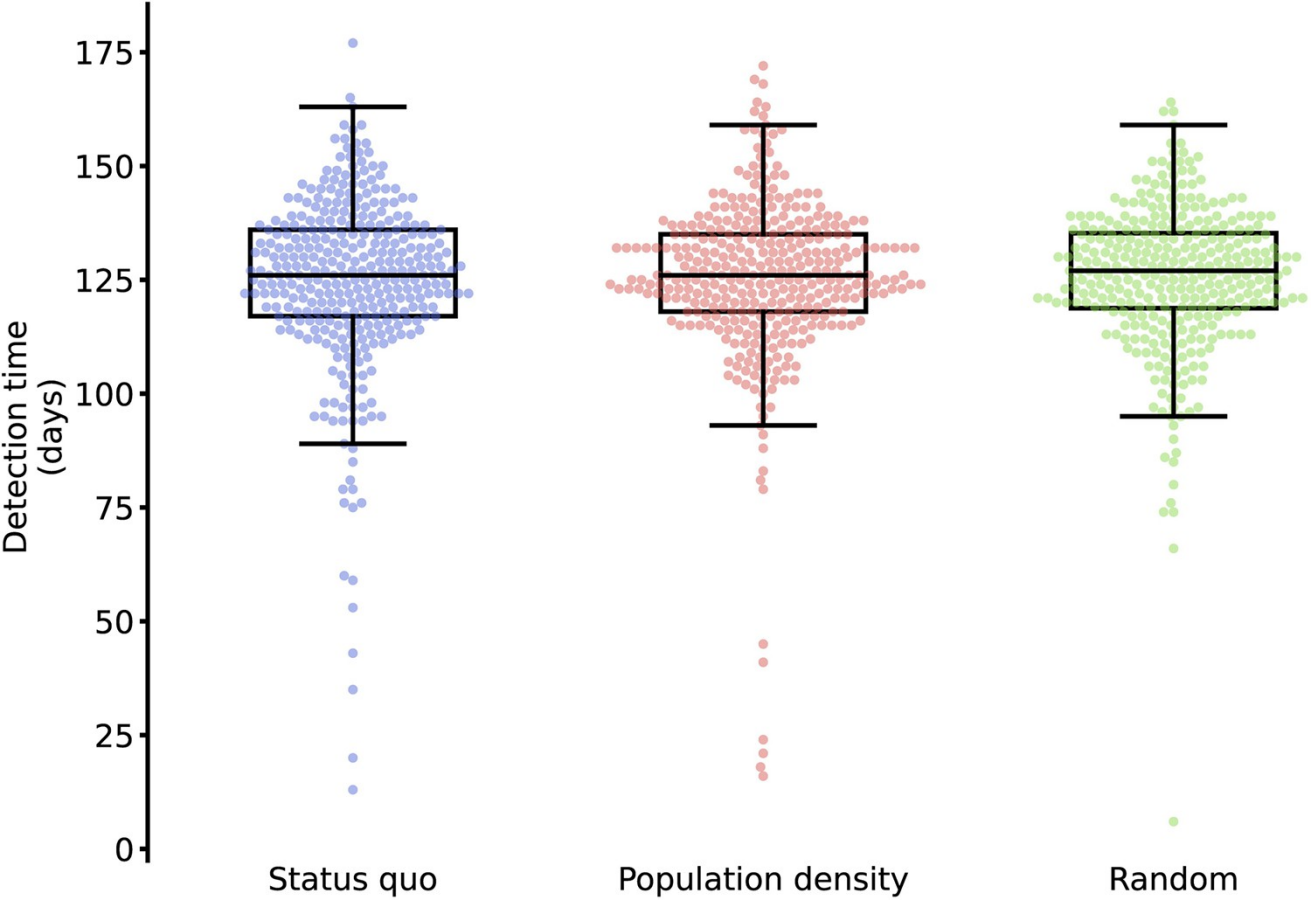
Distribution of detection times by geographic sampling strategy. Points depict the time between variant introduction and detection in days for the scenarios where tests are sampled geographically according to the baseline testing strategy, proportionally to population size, or randomly across New York City (at variant introduction 50 days after the prior variant, 30% of baseline test volume, and 10% sequencing rate). Boxes and whiskers depict the minimum, lower 25%, median, upper 75%, and maximum detection times.

**Fig 4:**
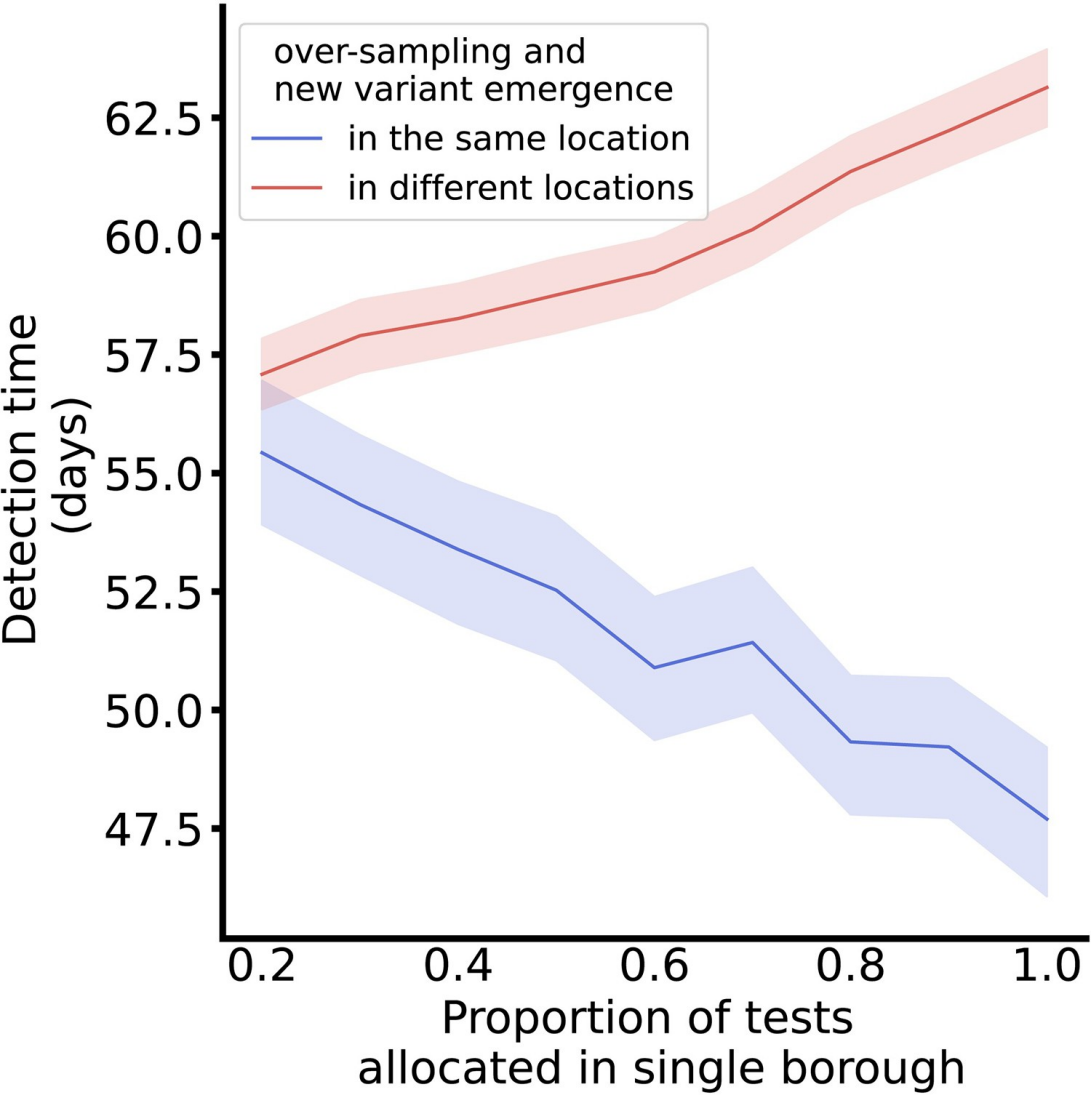
Detection time by proportion of tests allocated in a single location. Lines depict the average detection time for scenarios where between 20% and 100% of tests are sampled from a single location, and the remaining tests are evenly distributed across the remaining locations by population size. The lines distinguish between scenarios where the variant emerged in the primary allocation location, i.e., test over-sampling and emergence occurred in the same location (blue), and scenarios where the variant emerged in one of the other locations, i.e., test over-sampling and emergence occurred in different locations (red). Shaded areas depict the 95% simulation interval for the detection time.

Relative to the baseline volume and distribution of testing and sequencing in NYC (the “baseline” testing and sequencing strategy), detection times were similarly distributed when test volumes were allocated to be (a) proportional to the population density or (b) uniformly at random across locations (**Fig 3**). This similarity across geographic sampling strategies was unaffected by the outcome measure used as well as the timing and location of the new variant’s introduction. However, the geographic sampling strategy affected detection outcomes if the introduction location of the new variant was oversampled. Allocating a greater proportion of tests in a single location reduced detection times and cumulative infections of variants emerging in that location but increased detection times of variants that first appeared elsewhere (**Fig 4**). The size of the targeting effect was inversely correlated with total mobility and outward mobility of the different boroughs (**S6 and S7 Figs**).

### Emergence context

**Fig 5:**
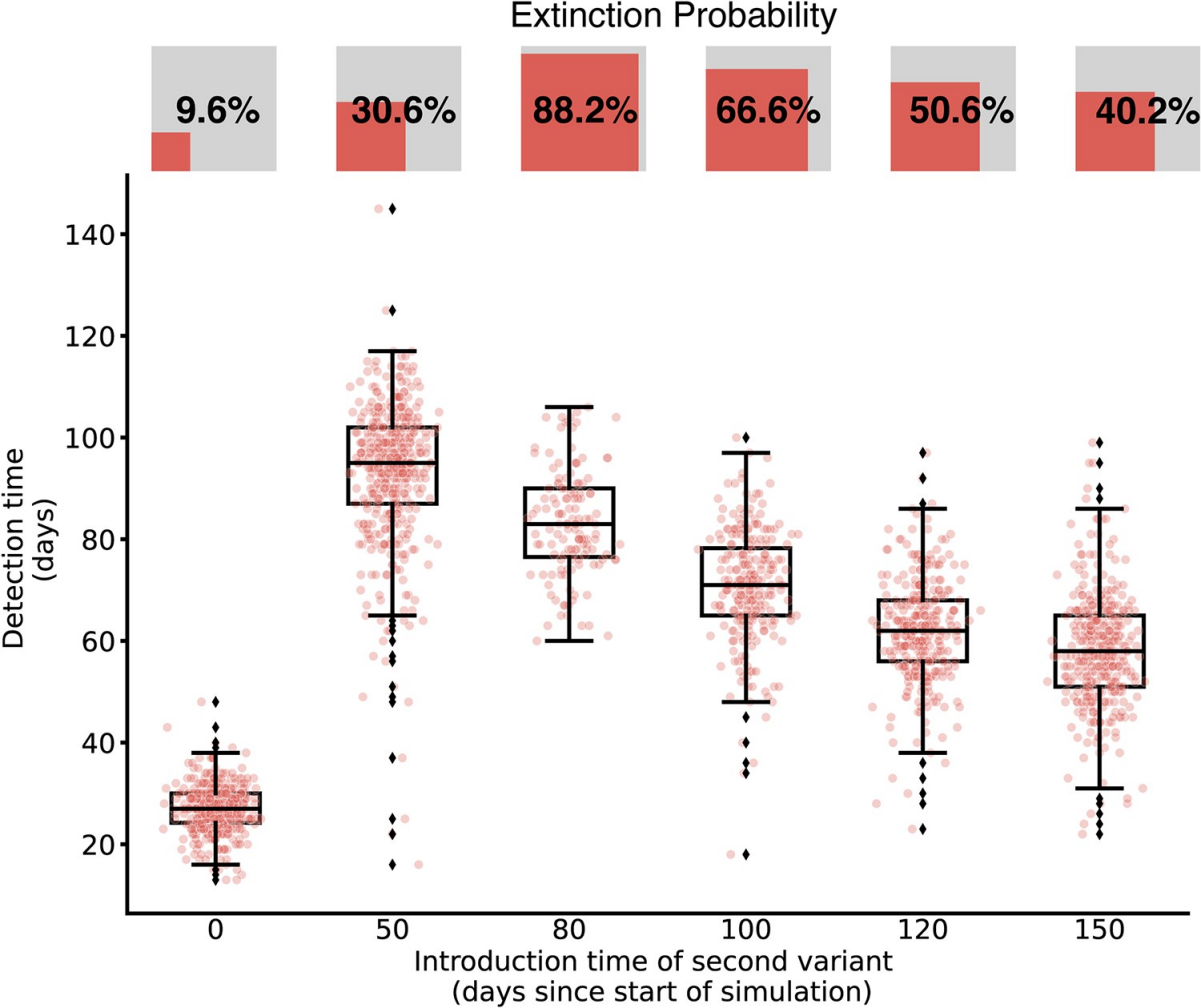
Detection time of a novel variant across introduction times. Points depict the time between variant introduction and detection in days for different introduction times (with baseline distribution of tests, 30% of baseline test quantity, and sequencing rate 10%). Points are jittered horizontally to help visualize the distribution. Boxes and whiskers depict the minimum, lower 25%, median, upper 75%, and maximum detection times. The extinction probability for each scenario is depicted using inset squares, where the relative area of the red square is proportional to the extinction probability.

We compared introduction times of the new variant as an approximation for varying background prevalence of the previously circulating variant and the population susceptibility to infection. When the second, more transmissible variant was introduced into a fully susceptible population together with the first variant (at *t* = 0), the second variant was more likely to dominate due to its increased transmissibility. Under this scenario, the extinction probability of the second variant (defined as the likelihood that a variant will cause no more than 10 infections) was only 9.6% under the baseline sampling strategy. Both variants generally persisted through the duration of the simulation, though the second variant caused more infections. Consequently, at a *t = 0* introduction time, the second variant was detected in under 33 days in 95% of simulations. If the second variant was introduced after the peak of the first variant’s outbreak (at *t* = 80 or *t* = 100), the second variant had a high probability of extinction (88.2 and 66.6%, respectively), and if it persisted, it was detected later (at least 56 and 37 days after introduction in 95% of simulations, respectively). As the time interval between the first variant’s peak and the second variant’s introduction increased (e.g. from *t* = 80 to *t* = 150) and immunity from infection with the first variant waned, detection times and extinction probabilities declined again. The greatest range of disease dynamics and consequently detection times was observed when the second variant was introduced just before the peak of the first variant (at *t* = 50), with detection times ranging from 16 to 145 days (**Fig 5**).

The introduction location did not significantly impact the detection time or cumulative disease burden across the city (**S8 Fig**) but did influence where infections occurred. The number of infections was highest in locations with the highest mobility connectivity to the emergence location, which was either the introduction location itself or other locations, depending on the mobility matrix. Emergence in Staten Island, for example, produced infections primarily within Staten Island, while emergence in Manhattan led to a high number of infections in Brooklyn and Queens (**S5 Fig**).

### Variant characteristics

**Fig 6:**
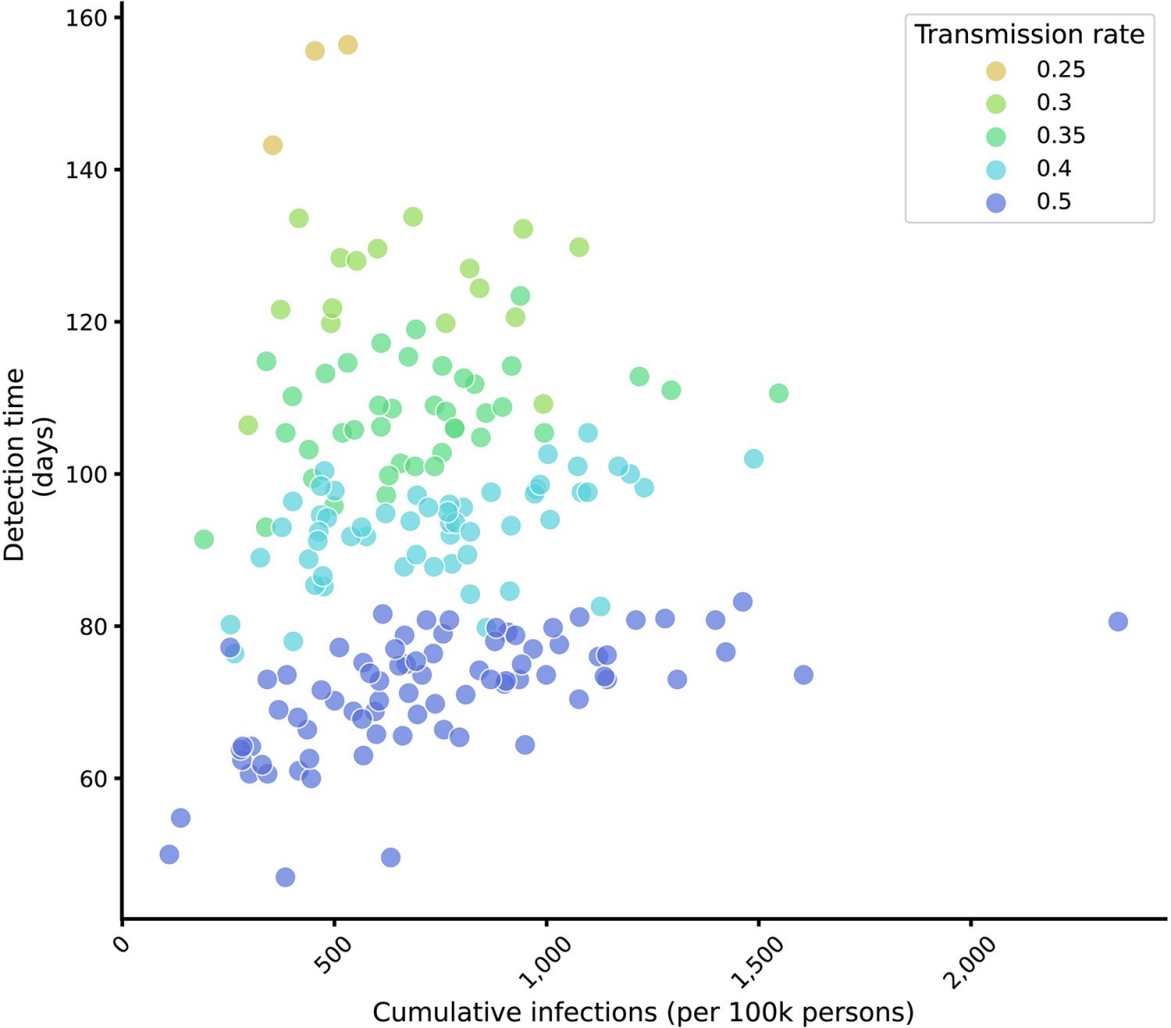
Detection time by cumulative infections for different transmission rates. Points depict the mean detection time and cumulative number of infections upon detection, averaged across 100 simulations of each introduction location, for each transmission probability from 0.25–0.5, represented by different colors (at variant introduction 50 days after the prior variant, baseline distribution of tests, 30% of baseline test quantity, and sequencing rate 10%). The baseline transmission rate of the pre-existing variant is β = 0.2.

We compared variants with different levels of transmissibility, varying the probability of infection given an infectious contact from *β* = 0.25 to *β* = 0.5 (contrasting with the transmissibility of the first variant of *β* = 0.2). This transmission parameter affected the disease dynamics, with more transmissible variants spreading more quickly, leading to earlier detection. All transmission rates yielded a wide range of cumulative infections at detection time (**Fig 6**).

### Comparison of surveillance and emergence characteristics

We estimated the relative impact of all factors on detection times in a multivariable regression model (**S2 Table**). Of the surveillance characteristics, raising sequencing proportions by 1 percentage point decreased detection time by 44 days (p<0.0001) and infections by 502 cases per 100,000 persons (p<0.0001). A 1 percentage point increase in per-capita test rates reduced detection times by 13 days (p<0.0001) and infections by 124 cases per 100,000 persons (p<0.0001). Shifting to the random or density-based strategy did not result in significant changes in detection speed or total disease burden. Emergence context also had a significant impact on detection, with a 0.1 percentage point increase in transmissibility of the novel variant led to a 60-day reduction in detection time (p<0.0001) and 133 additional infections per 100,000 persons (p<0.0001) at detection time.

## Discussion

This study provides an assessment of testing and sequencing strategies for the detection of new SARS-CoV-2 variants to help inform genomic surveillance policies. We considered varying quantities and distributions of resources within a wide range of potential settings for variant emergence and assessed how they influenced variant detection speed and the undetected disease burden. Our results confirmed that variant detection is governed by both the surveillance strategy and the epidemic dynamics in which the new variant arises [18].

While raising both sequencing proportions and testing rates reduced detection times and undetected infections, these improvements were driven primarily by the increased number of sequenced samples, which increase with rising rates of either testing or sequencing. This finding contributes to our understanding of how surveillance systems can be designed to optimize detection, building on existing research which demonstrated that testing volume should be sufficiently high to ensure that samples are representative of all COVID-19 infections (e.g., through increasing the number of sentinel sites or approximating population random sampling [11]).

The relative transmissibility of the new variant as well as the timing of its emergence influenced both its speed of spread and survival probability, which in turn affected the detection speed and undetected disease burden (**Figs 5 and 6**). This result is consistent with and expands on observations of lineage-dependent effects of down-sampling genomic sequencing data [12] as well as more explicit calculations of variant-specific biological and logistical biases in sample size calculations for variant detection [9].

Targeted testing in locations with high positivity rates reduces the number of undetected COVID-19 infections [19]. Our simulations showed that geographic targeting of locations with likely variant introduction (e.g., ports of entry) or emergence (e.g., hospitals) can also improve detection outcomes. The connectivity of the introduction location did not impact detection times but did affect where infections occurred before variant detection (**S7 and** S8 **Figs**). Variants that emerged among residents of boroughs with more inward and outward mobility produced more infections in other boroughs. In our simulations, a variant first appearing in a resident of Manhattan, for example, caused more infections on average in Brooklyn and Queens than in Manhattan itself, likely due to a combination of the high outward mobility and low inward mobility of Manhattan (**S7 Fig**) as well as the boroughs’ relative population sizes. Failing to adequately sample locations near emergence or those highly connected to emergence locations could lead to a disproportionate number of infections in those locations.

Changing the geographic distribution of testing, without specifically targeting emergence locations, had little impact on detection outcomes in our simulations, though this may be driven by the random sampling assumption in this case study of NYC. Prior research has shown that sampling from few sentinel sites with low testing volume negatively impacts variant detection, relative to random population-wide sampling [11]. Geographic distribution of testing may therefore be more relevant in contexts where random sampling is not yet attainable. Further, competing public health objectives—including fairness and equitable access to care—must be balanced to inform how testing capacity is allocated across a city. Distributing limited capacity according to a density-based strategy may help achieve equity and, according to the results of our analysis, should not significantly affect variant detection speed relative to the baseline allocation. In implementing a given surveillance strategy, decision-makers must weigh benefits of variant detection and indirect impacts on other public health objectives (such as disease control effects of increased testing) against the costs associated with both testing and sequencing, which are highly dependent on local contexts, such as the available capacity for sequencing or pooled testing [20,21].

The number of undetected infections varied widely for a given transmission rate, even at fixed detection times (**Fig 6**). This result demonstrated the challenge of understanding the epidemiologic scenario on discovery of a new variant and the need for combining pathogen genome sequencing with other forms of surveillance. More work is also needed to understand whether optimal surveillance strategies differ if the primary objective is monitoring or detecting variants and how to position genomic surveillance within the broader landscape of sometimes competing public health objectives.

The model in this study was designed to be simple, while accounting for the most important factors affecting testing and sequencing, and to help attain a qualitative understanding of which parameters influence detection times and the number of infections at the time of first detection. The model was not fit to disease dynamics observed for any given SARS-CoV-2 variant, but rather evaluated relative changes in detection speed and burden for different surveillance strategies, epidemiologic settings, and variant characteristics. Consequently, the simulation results, such as the detection times, should not be interpreted as predictions. Specific simplifications included the modeling of single introductions of a novel variant, rather than accounting for multiple introductions or several variants. We assumed homogeneous mixing within locations and did not account for age structure and other demographic factors, social networks, or social determinants of health. SARS-CoV-2 infection risk varies across socioeconomic and demographic groups, due in part to variability in the average number of contacts, vaccine uptake, long- and short-distance mobility, comorbidities linked to more severe disease outcomes, and other social factors [22–24]. While we incorporated neighborhood-level variations in movement, we did not include within-neighborhood heterogeneity or between-neighborhood variation in social determinants of health. Increased data stratified by socioeconomic and demographic factors and continued research will be critical to explaining the experience of disparities in health outcomes during the COVID-19 pandemic. In particular, we still lack a complete understanding of how social and demographic heterogeneities influence where new variants emerge, how they spread, and consequently when and where they are detected. This data and research are needed to inform future prevention and response efforts that also advance health equity. We took a simplified view of genomic surveillance processes. We assumed random sampling of positive tests and did not account for variations in specimen quality across testing sites or in access to testing, which may cloud estimates of the prevalence of circulating variants [9]. In this sense, our model takes an idealized view of our capacity to sample randomly from the population. Our model simulated the spread of two distinct variants, though results can be expanded to multiple variants that are introduced with small numbers of initial cases into distinct population subgroups.

Emerging empirical evidence on genomic surveillance of SARS-CoV-2 variants has allowed public health agencies to provide guidance on sampling strategies to detect and monitor variants, though more research is needed to anticipate the impact of these strategies under as yet unseen epidemiologic settings. This modeling study aimed to contribute to these ongoing efforts to assess variant detection strategies, by simulating detection outcomes for varying testing and sequencing rates in NYC. Our results highlight the importance of sequencing and geographic targeting for variant detection and showed that the timing of emergence and variant properties can impact detection as much as changes to surveillance strategies. To detect new variants quickly, genomic sequencing should be prioritized, ensuring representative sampling and targeted testing, and interpreting results in light of the epidemiological context.

## Supporting information

S1 Text Materials and Methods. (DOCX)

S1 Fig Model structure. (TIF)

S2 FigMixing among locations.Panel A illustrates how the contact matrix is derived from the mobility matrix. Contact between residents of locations *i* and *j* is defined by the average number of contacts per person, *μ*_*contacts*_, and the probability of residents of location *i* encountering a resident of location *j*, which is in turn defined by the movement of residents of locations *i*, *j* to any other location *k*, *M*_{*i*→*k*}_, *M*_{*j*→*k*}_ and the total amount of movement to that location *k* from residents of any location *l*, ∑_*l*_ *M*_{*l*→*k*}_. Panel B illustrates how the contact matrix influences transmission among locations in the model. The likelihood that a resident of location *i* moves from the susceptible to the infectious state is defined by the level of contact with each other location, *k*_*ij*_, and within the same location, *k*_*ii*_, as well as the proportion of individuals in those respective locations that are infectious, $\frac{I_{i}}{N_{i}},\frac{I_{j}}{N_{j}}$. Infections are tracked by location of residence.(TIFF)

S3 Fig Example of test rates at the borough level. Boroughs are colored by the proportion of the population that is tested each week under the baseline (A), density-based (B), and random (C) sampling strategy. Copyright: OpenStreetMap, openstreetmap.org/copyright.
(DOCX)

S4 Fig Sensitivity analysis of fixed sequencing capacity. Lines depict the mean duration between variant introduction and detection in days (A) as a function of daily testing volume, colored by the maximum sequencing volume, and (B) as a function of maximum sequencing volume, colored by the test volume.
(TIF)

S5 Fig Cumulative infections by borough for introduction locations Manhattan and Staten Island. Points depict the number of cumulative infections in each borough at detection time (at variant introduction 50 days after the prior variant, baseline distribution of tests, 30% of baseline test quantity, and sequencing rate 10%). Boxes and whiskers depict the minimum, lower 25%, median, upper 75%, and maximum cumulative infections.
(TIF)

S6 Fig Change in detection time by increasing proportion of tests allocated in a single location, by introduction location. Lines depict and ribbons the average and 95% simulation interval of the change in detection time for scenarios where the proportion of tests allocated to a single location increases from 20% to between 30% and 100%. The sub-plots distinguish between scenarios where the variant emerged in the primary allocation location, i.e., test over-sampling and emergence occurred in the same location (left), and scenarios where the variant emerged in one of the other locations, i.e., test over-sampling and emergence occurred in different locations (right).
(TIF)

S7 Fig Rankings of boroughs by mobility volume. Boxes are shaded by the rank of each borough’s level of connectivity according to total mobility (first row), within-borough mobility (second row), and between-borough mobility (third row), where darker shades of blue represent higher mobility.
(TIF)

S8 Fig Detection times by introduction location. Points depict the detection time in days for each introduction location (at variant introduction 50 days after the prior variant, baseline distribution of tests, 30% of baseline test quantity, and sequencing rate 10%). Boxes and whiskers depict the minimum, lower 25%, median, upper 75%, and maximum detection times.
(TIF)

S9 Fig Comparing contact rates. Detection time (A) and cumulative infections at detection time (B) for varying numbers of average contacts per person for introduction time t = 0.
(TIF)

S10 Fig Comparing incubation periods. Detection time (A) and cumulative infections at detection time (B) for varying durations of incubation periods of novel variant (3, 5, 7 days) and a fixed incubation period of 5 days of the base variant.
(TIF)

S1 Table Parameters. (DOCX)

S2 Table Multivariable regression results. (DOCX)
