# Supplementary material for: Surveillance strategies for the detection of new pathogen variants across epidemiological contexts
Source: PLoS Comput Biol. 2024 Sep 5;20(9):e1012416. doi: 10.1371/journal.pcbi.1012416 (PMC11407617; doi:10.1371/journal.pcbi.1012416)
Supplement: S1 Text — (DOCX) [file pcbi.1012416.s001.docx]

**S1. Materials and Methods**

***Data***

*Mobility*

Through its Facebook Data for Good initiative, Meta provides aggregated and anonymized movement data from users who have enabled location sharing (1). It is available in 8-hour intervals and with a maximum geographic resolution of 600-by-600m tile sizes, capturing from 75 to 173 thousand user movements per borough per month during our study period. We leveraged this data to compute the average share of users moving between geographic tiles on a given day, aggregated to boroughs. This percentage was rescaled to the population size of each location, under the assumption that Facebook users who activate location sharing are representative of NYC residents, which may not be true in practice and may limit our ability to accurately capture human movement across the city. The movements were captured in the raw mobility matrix $M$, where an entry $M_{\{i->j\}}$indicates the number of movements from location $i$ to location $j$. We then computed a contact matrix from the mobility matrix under the assumption of homogeneous mixing. The probability that a resident of location $i$ has contact with a resident of location $j$ in any location $k$ was defined as: ​

$c_{\{i,j\}}=\sum_{k} \frac{M_{\{i->k\}}M_{\{j->k\}} \mu_{contacts}}{\sum_{l} M_{\{l->k\}}}$

Where $\mu_{contacts}$is the average number of contacts per person, $M_{\{i->k\}}$ is the number of individuals moving from location $i$ to location $k$, and $\sum_{l} M_{\{l->k\}}$ is the sum of all individuals moving to location $k$. This contact matrix determined the coupling strength of two locations in the mathematical model, and therefore influenced the likelihood for an infection to spread between the two locations. In the main analysis, we assumed 4 average contacts per person. In sensitivity analyses, we considered 2 and 8 average contacts (Figure S9).

*Testing and Sequencing Rates*

Weekly COVID-19 testing rates and sequencing rates by MODZCTA are published by the NYC Department of Health and Mental Hygiene [(16)](https://www.zotero.org/google-docs/?jmyyCW). The baseline test rate was calculated as the daily average from December 2020 to November 2021.

***Compartmental Model Structure***

We implemented a multi-patch, two-variant stochastic compartmental model that builds upon the basic structure of an SEIRS model. Exposure, testing, sequencing, isolation, recovery, waning (cross-)protection, and reinfection were treated as stochastic events. The model explicitly incorporated human mobility, testing and sequencing rates, as well as test sensitivity and specificity. It also included control measures to account for the fact that testing strategies may impact disease dynamics if infectious individuals reduce their contacts upon receiving a positive test result.

The model structure is presented in Figure S1 and described in more detail below. The corresponding parameters are listed in Supplemental Table 1. Susceptible ($S$) individuals may be exposed ($E$) to one of two variants, represented by subscripts 1 and 2, or they may isolate if they receive a false positive test result ($S_{q}$). After a latent period, an exposed individual moves to one of five infectious compartments depending on their reporting and isolation outcome: an infection may be unreported ($I_{U}$), for example if it is asymptomatic or detected only via an at-home test. A positive case may be detected at an official test center ($I_{T}$), leading the person to isolate ($I_{Tq}$) or not ($I_{Tnq}$). Finally, the positive test may also be sequenced ($I_{G}$), again separating individuals by their isolation status ($I_{Gq}, I_{Gnq}$), where isolation reduces the transmission probability by $\left( 1-\theta\right)$. After recovery, individuals have temporary full protection against reinfection with either variant while they remain in compartments $R_{U}, R_{T}, R_{G}$. Full protection wanes over time at variant-specific rates, making individuals susceptible to reinfection with one ($S_{1\{1\}}, S_{2\{1\}}, S_{1\{2\}}, S_{2\{2\}}$) or both variants ($S_{12\{1\}}, S_{12\{2\}}$). The model incorporates a variant-specific leaky immunity parameter ($a_{11}, a_{12}, a_{21}, a_{22}$), which defines the reduction in the probability of reinfection after full protection has waned.

$$\frac{dS}{dt}= \epsilon S_{q} - p_{t}(1-p_{TP}) p_{q} S - b_{1}kI_{1}S - b_{2}kI_{2}S$$

$$\frac{dS_{q}}{dt}= p_{t}(1-p_{TP}) p_{q} S - \epsilon S_{q}$$

*Variant 1:*

$$\frac{dE_{1}}{dt} = b_{1}kI_{1}S +a_{1\{1\}}b_{1}kI_{1}{(S}_{1\{1\}}+S_{12\{1\}}) +a_{1\{2\}}b_{1}kI_{1}{(S}_{1\{2\}}+S_{12\{2\}}) -\frac{1}{L_{1}}E_{1}$$

$$\frac{dI_{U1}}{dt}=\frac{1}{L_{1}}E_{1} [(1-p_{t})+p_{t}p_{FN}] - \frac{1}{D_{1}}I_{U1}$$

$$\frac{dI_{Tq1}}{dt}=\frac{1}{L_{1}}E_{1} [p_{t}(1-p_{g})p_{TP}p_{q}] - \frac{1}{D_{1}}I_{Tq1}$$

$$\frac{dI_{Tnq1}}{dt}=\frac{1}{L_{1}}E_{1} [p_{t}(1-p_{g})p_{TP}{(1- p}_{q})] - \frac{1}{D_{1}}I_{Tnq1}$$

$$\frac{dI_{Gq1}}{dt}=\frac{1}{L_{1}}E_{1} [p_{t} p_{g} p_{TP}p_{q}] - \frac{1}{D_{1}}I_{Gq1}$$

$$\frac{dI_{Gnq1}}{dt}=\frac{1}{L_{1}}E_{1} [p_{t} p_{g} p_{TP}{(1- p}_{q})] - \frac{1}{D_{1}}I_{Gnq1}$$

$$\frac{dR_{U1}}{dt}=\frac{1}{D_{1}}I_{U1} - R_{U1}(w_{1\{1\}}+w_{2\{1\}}-w_{1\{1\}}w_{2\{1\}})$$

$$\frac{dR_{T1}}{dt}=\frac{1}{D_{1}}{(I}_{Tq1}+I_{Tnq1}) - R_{T1}(w_{1\{1\}}+w_{2\{1\}}-w_{1\{1\}}w_{2\{1\}})$$

$$\frac{dR_{G1}}{dt}=\frac{1}{D_{1}}{(I}_{Gq1}+I_{Gnq1}) - R_{G1}(w_{1\{1\}}+w_{2\{1\}}-w_{1\{1\}}w_{2\{1\}})$$

$$\frac{dS_{1\{1\}}}{dt}= w_{1\{1\}}(1-w_{2\{1\}})(R_{U1}+ R_{T1} + R_{G1}) -a_{1\{1\}}b_{1}kI_{1}S_{1\{1\}}$$

$$\frac{dS_{12\{1\}}}{dt}= w_{1\{1\}}w_{2\{1\}} (R_{U1}+ R_{T1} + R_{G1}) -a_{1\{1\}}b_{1}kI_{1}S_{12\{1\}}-a_{2\{1\}}b_{2}kI_{2}S_{12\{1\}}$$

$$\frac{dS_{2\{1\}}}{dt}= w_{2\{1\}}(1-w_{1\{1\}})(R_{U1}+ R_{T1} + R_{G1}) -a_{2\{1\}}b_{2}kI_{2}S_{2\{1\}}$$

*Variant 2:*

$$\frac{dE_{2}}{dt} = b_{2}kI_{2}S +a_{2\{1\}}b_{2}kI_{2}{(S}_{2\{1\}}+S_{12\{1\}}) +a_{2\{2\}}b_{2}kI_{2}{(S}_{2\{2\}}+S_{12\{2\}}) -\frac{1}{L_{2}}E_{2}$$

$$\frac{dI_{U2}}{dt}=\frac{1}{L_{2}}E_{2} [(1-p_{t})+p_{t}p_{FN}] - \frac{1}{D_{2}}I_{U2}$$

$$\frac{dI_{Tq2}}{dt}=\frac{1}{L_{2}}E_{2} [p_{t}(1-p_{g})p_{TP}p_{q}] - \frac{1}{D_{2}}I_{Tq2}$$

$$\frac{dI_{Tnq2}}{dt}=\frac{1}{L_{2}}E_{2} [p_{t}(1-p_{g})p_{TP}{(1-p}_{q})] - \frac{1}{D_{2}}I_{Tnq2}$$

$$\frac{dI_{Gq2}}{dt}=\frac{1}{L_{2}}E_{2} [p_{t} p_{g} p_{TP}p_{q}] - \frac{1}{D_{2}}I_{Gq2}$$

$$\frac{dI_{Gnq2}}{dt}=\frac{1}{L_{2}}E_{2} [p_{t} p_{g} p_{TP}{(1- p}_{q})] - \frac{1}{D_{2}}I_{Gnq2}$$

$$\frac{dR_{U2}}{dt}=\frac{1}{D_{2}}I_{U2} - R_{U2}(w_{1\{2\}}+w_{2\{2\}}-w_{1\{2\}}w_{2\{2\}})$$

$$\frac{dR_{T2}}{dt}=\frac{1}{D_{2}}{(I}_{Tq2}+I_{Tnq2}) - R_{T2}(w_{1\{2\}}+w_{2\{2\}}-w_{1\{2\}}w_{2\{2\}})$$

$$\frac{dR_{G1}}{dt}=\frac{1}{D_{2}}{(I}_{Gq2}+I_{Gnq2}) - R_{G2}(w_{1\{2\}}+w_{2\{2\}}-w_{1\{2\}}w_{2\{2\}})$$

$$\frac{dS_{1\{2\}}}{dt}= w_{1\{2\}}(1-w_{2\{2\}})(R_{U2}+ R_{T2} + R_{G2}) -a_{1\{2\}}b_{1}kI_{1}S_{1\{2\}}$$

$$\frac{dS_{12\{2\}}}{dt}= w_{1\{2\}}w_{2\{2\}} (R_{U2}+ R_{T2} + R_{G2}) -a_{1\{2\}}b_{1}kI_{1}S_{12\{2\}}-a_{2\{2\}}b_{2}kI_{2}S_{12\{2\}}$$

$$\frac{dS_{2\{2\}}}{dt}= w_{2\{2\}}(1-w_{1\{2\}})(R_{U2}+ R_{T2} + R_{G2}) -a_{2\{2\}}b_{2}kI_{2}S_{2\{2\}}$$

where $I_{1}=I_{U1}+I_{Tnq1}+I_{Gnq1}+\theta*({I_{Tq1}+I}_{Gq1})$ and $I_{2}=I_{U2}+I_{Tnq2}+I_{Gnq2}+{\theta*(I}_{Tq2}+I_{Gq2})$

Where $p_{t}$ is the test rate, $p_{g}$ is the sequencing rate, $p_{q}$ is the control measure compliance rate, $p_{TP}$ is the true positive rate, $a_{x\{y\}}$ is the leaky immunity parameter, $w_{x\{y\}}$ is the waning full immunity parameter, $b$is the probability of infection given contact, $L$ is the average duration of the latent period, $D$ is the average duration of the infectious period, $k$ is the contact matrix, and $\epsilon$ is the rate of ending control measures if false positive. The effective contact rate is therefore jointly defined by the average number of contacts, $\mu_{contacts}$, the probability of transmission given an infectious contact, $b_{1},b_{2}$, the number of infectious individuals, $I_{1},I_{2}$, as well as their level of adherence to control measures,$p_{q}$, and relative transmissibility while adhering to control measures, $\theta$.

***Simulations***

*Geographic Test Distribution*

We implement the following primary strategies for testing allocation. Figure S3 shows an example of the test rates under each scenario.

- Baseline: Tests were allocated according to the historical NYC strategy, computed as the average allocation and daily test quantity between December 2020 and November 2021.
- Population density-based allocation: Tests were distributed proportionally to population sizes of each location, so that each location had the same test rate.
- Random allocation: The available tests were distributed randomly across locations. This typically resulted in higher per-capita test rates in less densely populated areas.

*Test Volume*

We varied the quantity of available tests from 5% to 400% of the NYC average of 7,184 tests per day (87 tests per 100,000 persons). Changing the quantity of available tests may be understood as either increased capacity or reduced reporting, for example due to a shift toward home testing. From February 2020 to September 2021, an estimated 75 percent of COVID-19 infections went unreported (13). With the emergence of the Omicron sub-variants BA.2.12 and BA.2.12.1, underreporting may be as high as 95 percent (14).

To investigate the role of the geographic test distribution, we considered “focused testing scenarios”, where individual locations are over-sampled. In these scenarios, 20-100% of all tests were sampled from a single location and the remaining tests were distributed across the remaining locations proportional to population size. We compared how detection outcomes differed when the new variant emerged in the over-sampled location *versus* one of the other locations.

*Sequencing volume*

The main analysis considered sequencing probabilities of 5-90% of all positive tests. The sequencing volume for each day of the simulation was therefore determined by the test volume and the number of infections. Raising test volume for a given epidemiologic scenario effectively raises sequencing volume at fixed sequencing probabilities.

In two sensitivity analyses, we sought to isolate the effect of testing and sequencing volumes by placing a cap on sequencing resources:

- Sensitivity analysis 1: At any time point, a fixed number of positive tests was sequenced. If there were fewer positive tests than sequencing resources (due to low prevalence), then all positive tests were sequenced. The maximum sequencing volume was reached when there was a sufficient quantity of positive tests, i.e., sufficiently high prevalence and test volume. The effective sequencing volume did not necessarily vary for different sequencing caps, if prevalence and/or test volume were low.
- Sensitivity analysis 2: A fixed number of tests were assigned as potentials for sequencing, akin to placing a stamp on a subset of test kits. If a test with a stamp was positive, the sample was sequenced. The maximum sequencing volume was achieved when all (stamped) tests were positive, which depended on the number of infections. The effective sequencing volume necessarily varied between the different sequencing caps.

*Variants*

The first variant was modeled to resemble the Delta SARS-CoV-2 variant with an effective transmission probability of 0.2. We then introduced a second variant, which was (i) more transmissible, (ii) had greater and faster immune evasion, or (iii) both. We considered effective transmission probabilities of 0.21-0.5.

*Context of variant emergence*

We simulated the emergence of the second variant at different locations and different times relative to the introduction of the first variant. The first variant was introduced with a single index case in each location, to simulate even spread across the city. The second variant was introduced with one index case in a single location, representing the location of residence of the index case. We simulated all possible introduction locations. Introduction times of the second variant varied from 0 to 150 days after the introduction of the first variant. The introduction times represent different contexts, because of varying prevalence of the first variant and varying numbers of susceptible individuals. We did not distinguish between variants that emerged within the city and those that were imported. The index case of the novel variant in our model could thus represent the first case of a newly emerging variant introduced to NYC from outside the city or a globally undetected variant that either emerged within NYC or was imported from outside before detection.

*Outcome measures*

For each simulation, we computed three primary outcome measures. The *time to detection* was defined as the number of days between the introduction and detection (first sequenced case) of the second variant. *Cumulative undetected infections* measured the total number of people who were exposed to (i.e., infected by) the second variant in NYC by the time the new variant was detected, including individuals who at detection time were in the latent phase prior to infectiousness, infectious, recovered, or susceptible to reinfection. Finally, we computed the *standard deviation of the cumulative undetected infections* across locations as an estimate of the geographic variation in disease burden.

**References**

1. Maas P. Facebook Disaster Maps: Aggregate Insights for Crisis Response & Recovery. In: Proceedings of the 25th ACM SIGKDD International Conference on Knowledge Discovery & Data Mining [Internet]. New York, NY, USA: Association for Computing Machinery; 2019 [cited 2022 Nov 22]. p. 3173. (KDD ’19). Available from: https://doi.org/10.1145/3292500.3340412

2. NYC Coronavirus Disease 2019 (COVID-19) Data [Internet]. NYC Department of Health and Mental Hygiene; 2022 [cited 2022 May 25]. Available from: https://github.com/nychealth/coronavirus-data

3. Kaine G, Greenhalgh S, Wright V. Compliance with Covid-19 measures: Evidence from New Zealand. PLOS ONE. 2022 Feb 9;17(2):e0263376.

4. Firth JA, Hellewell J, Klepac P, Kissler S, Kucharski AJ, Spurgin LG. Using a real-world network to model localized COVID-19 control strategies. Nat Med. 2020 Oct;26(10):1616–22.

5. Talic S, Shah S, Wild H, Gasevic D, Maharaj A, Ademi Z, et al. Effectiveness of public health measures in reducing the incidence of covid-19, SARS-CoV-2 transmission, and covid-19 mortality: systematic review and meta-analysis. BMJ. 2021 Nov 18;375:e068302.

6. Feikin DR, Higdon MM, Abu-Raddad LJ, Andrews N, Araos R, Goldberg Y, et al. Duration of effectiveness of vaccines against SARS-CoV-2 infection and COVID-19 disease: results of a systematic review and meta-regression. The Lancet. 2022 Mar 5;399(10328):924–44.

7. Davies NG, Klepac P, Liu Y, Prem K, Jit M, Eggo RM. Age-dependent effects in the transmission and control of COVID-19 epidemics. Nat Med. 2020 Aug;26(8):1205–11.

8. Bar-On YM, Flamholz A, Phillips R, Milo R. SARS-CoV-2 (COVID-19) by the numbers. eLife. 9:e57309.

9. Hay JA, Kissler SM, Fauver JR, Mack C, Tai CG, Samant RM, et al. Quantifying the impact of immune history and variant on SARS-CoV-2 viral kinetics and infection rebound: A retrospective cohort study. Rodriguez-Barraquer I, Ferguson NM, Azman A, editors. eLife. 2022 Nov 16;11:e81849.

10. Feehan DM, Mahmud AS. Quantifying population contact patterns in the United States during the COVID-19 pandemic. Nat Commun. 2021 Feb 9;12(1):893.

11. Miyahara R, Tamura K, Kato T, Nakazaki M, Otani K, Ko YK, et al. SARS-CoV-2 Variants and Age-Dependent Infection Rates among Household and Nonhousehold Contacts - Volume 29, Number 8—August 2023 - Emerging Infectious Diseases journal - CDC. [cited 2023 Nov 1]; Available from: https://wwwnc.cdc.gov/eid/article/29/8/22-1582_article

12. CDC. CDC Updates and Shortens Recommended Isolation and Quarantine Period for General Population. CDC [Internet]. 2021 Dec 27 [cited 2023 Nov 1]; Available from: https://www.cdc.gov/media/releases/2021/s1227-isolation-quarantine-guidance.html

13. CDC. Centers for Disease Control and Prevention. 2020 [cited 2022 May 26]. Cases, Data, and Surveillance. Available from: https://www.cdc.gov/coronavirus/2019-ncov/cases-updates/burden.html

14. Mandavilli A. Two new Omicron subvariants are spreading quickly in New York State. The New York Times [Internet]. 2022 Apr 13 [cited 2022 May 26]; Available from: https://www.nytimes.com/live/2022/04/13/world/covid-19-mandates-cases-vaccine
