# Supplementary figures and images for: Surveillance strategies for the detection of new pathogen variants across epidemiological contexts
Source: PLoS Comput Biol. 2024 Sep 5;20(9):e1012416. doi: 10.1371/journal.pcbi.1012416 (PMC11407617; doi:10.1371/journal.pcbi.1012416)

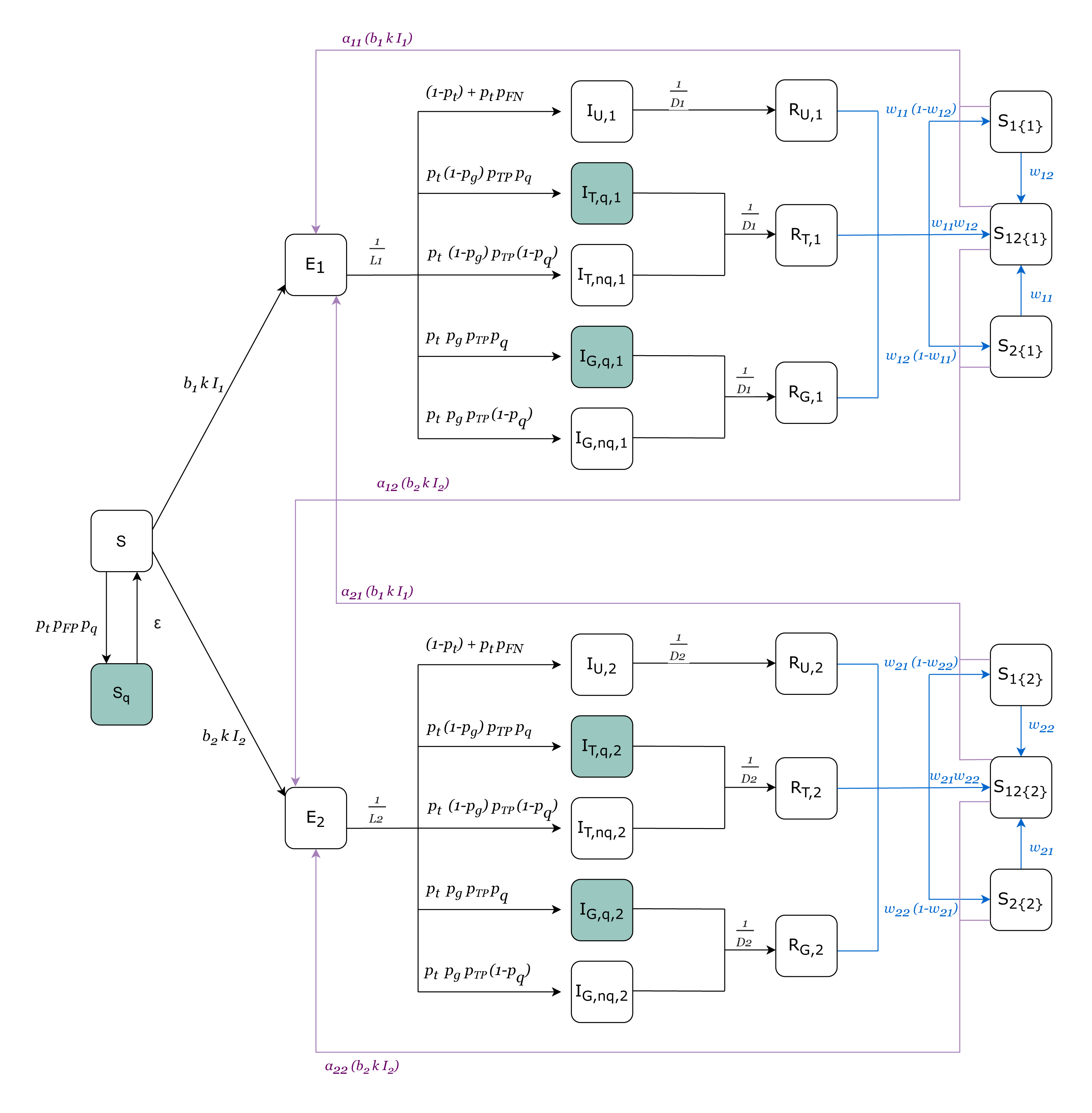

Supplement: S1 Fig — (TIF) [file pcbi.1012416.s002.tif]

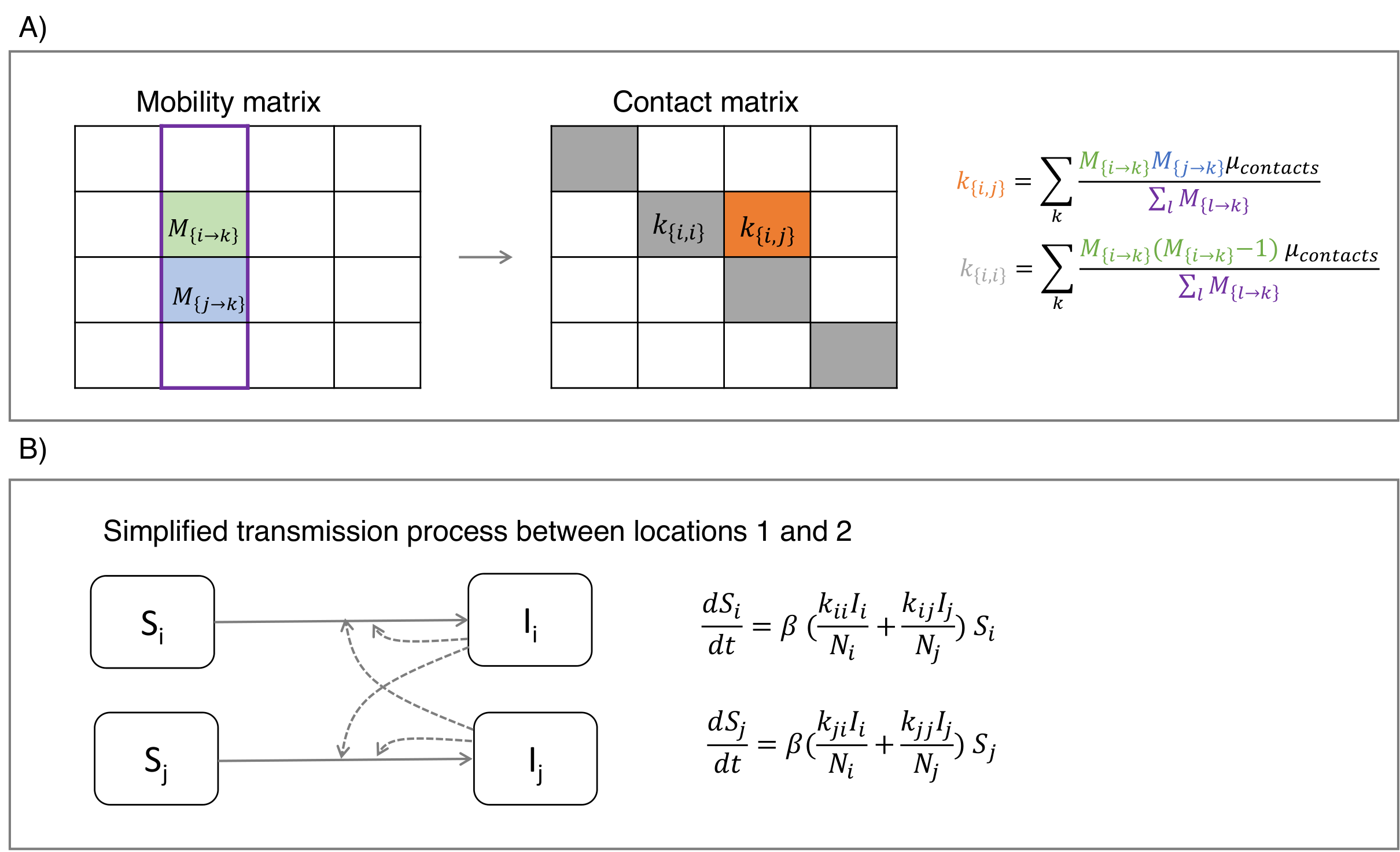

Supplement: S2 Fig — Panel A illustrates how the contact matrix is derived from the mobility matrix. Contact between residents of locations i and j is defined by the average number of contacts per person, μcontacts, and the probability of residents of location i encountering a resident of location j, which is in turn defined by the movement of residents of locations i, j to any other location k, M{i→k}, M{j→k} and the total amount of movement to that location k from residents of any location l, ∑l M{l→k}. Panel B illustrates how the contact matrix influences transmission among locations in the model. The likelihood that a resident of location i moves from the susceptible to the infectious state is defined by the level of contact with each other location, kij, and within the same location, kii, as well as the proportion of individuals in those respective locations that are infectious, IiNi,IjNj. Infections are tracked by location of residence. (TIFF) [file pcbi.1012416.s003.tiff]

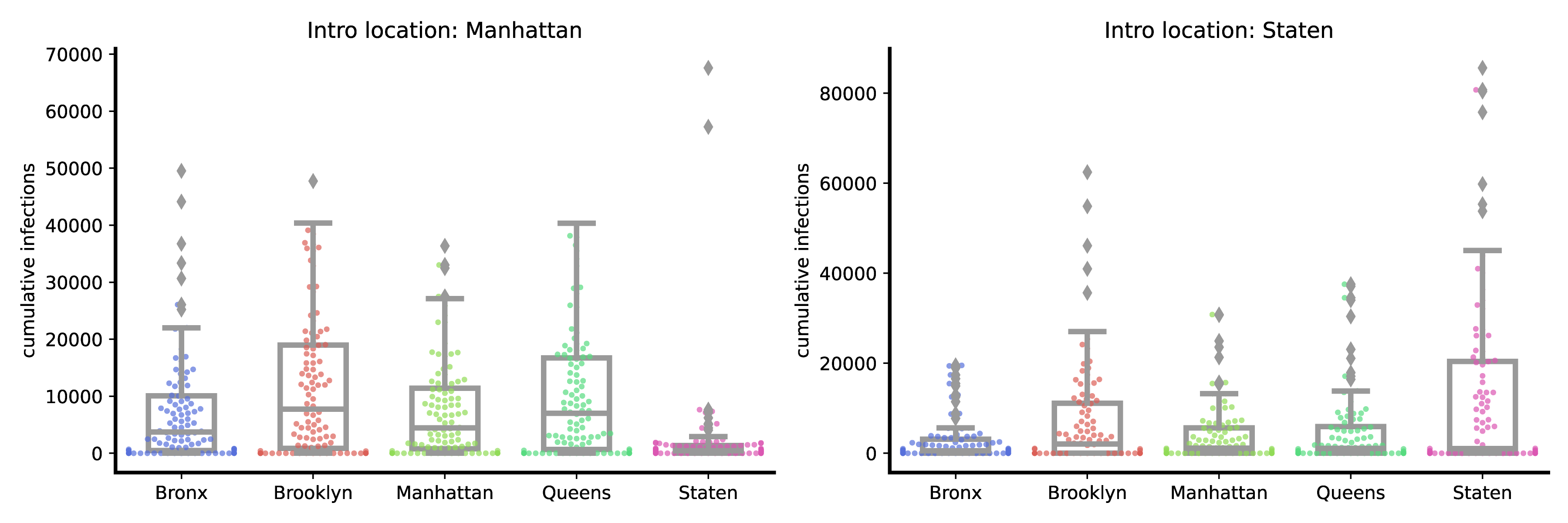

Supplement: S5 Fig — Points depict the number of cumulative infections in each borough at detection time (at variant introduction 50 days after the prior variant, baseline distribution of tests, 30% of baseline test quantity, and sequencing rate 10%). Boxes and whiskers depict the minimum, lower 25%, median, upper 75%, and maximum cumulative infections. (TIF) [file pcbi.1012416.s006.tif]

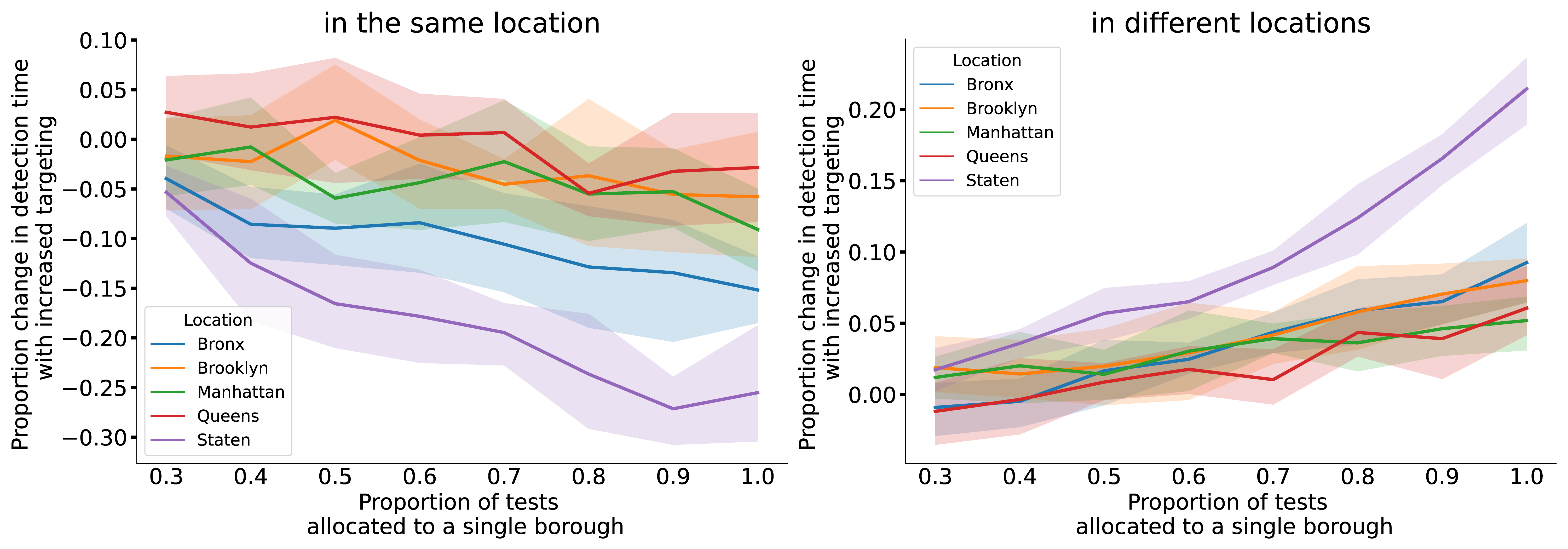

Supplement: S6 Fig — Lines depict and ribbons the average and 95% simulation interval of the change in detection time for scenarios where the proportion of tests allocated to a single location increases from 20% to between 30% and 100%. The sub-plots distinguish between scenarios where the variant emerged in the primary allocation location, i.e., test over-sampling and emergence occurred in the same location (left), and scenarios where the variant emerged in one of the other locations, i.e., test over-sampling and emergence occurred in different locations (right). (TIF) [file pcbi.1012416.s007.tif]

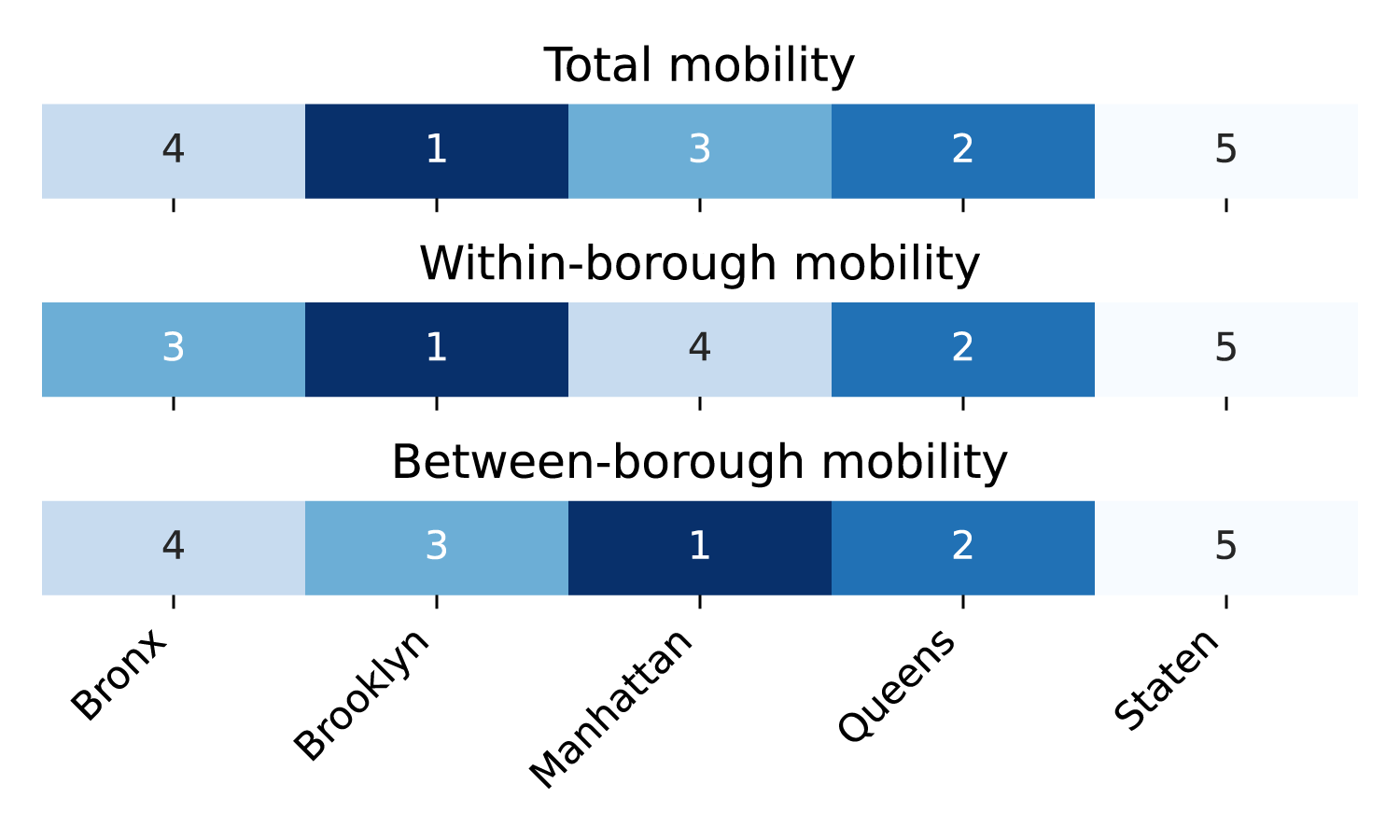

Supplement: S7 Fig — Boxes are shaded by the rank of each borough’s level of connectivity according to total mobility (first row), within-borough mobility (second row), and between-borough mobility (third row), where darker shades of blue represent higher mobility. (TIF) [file pcbi.1012416.s008.tif]

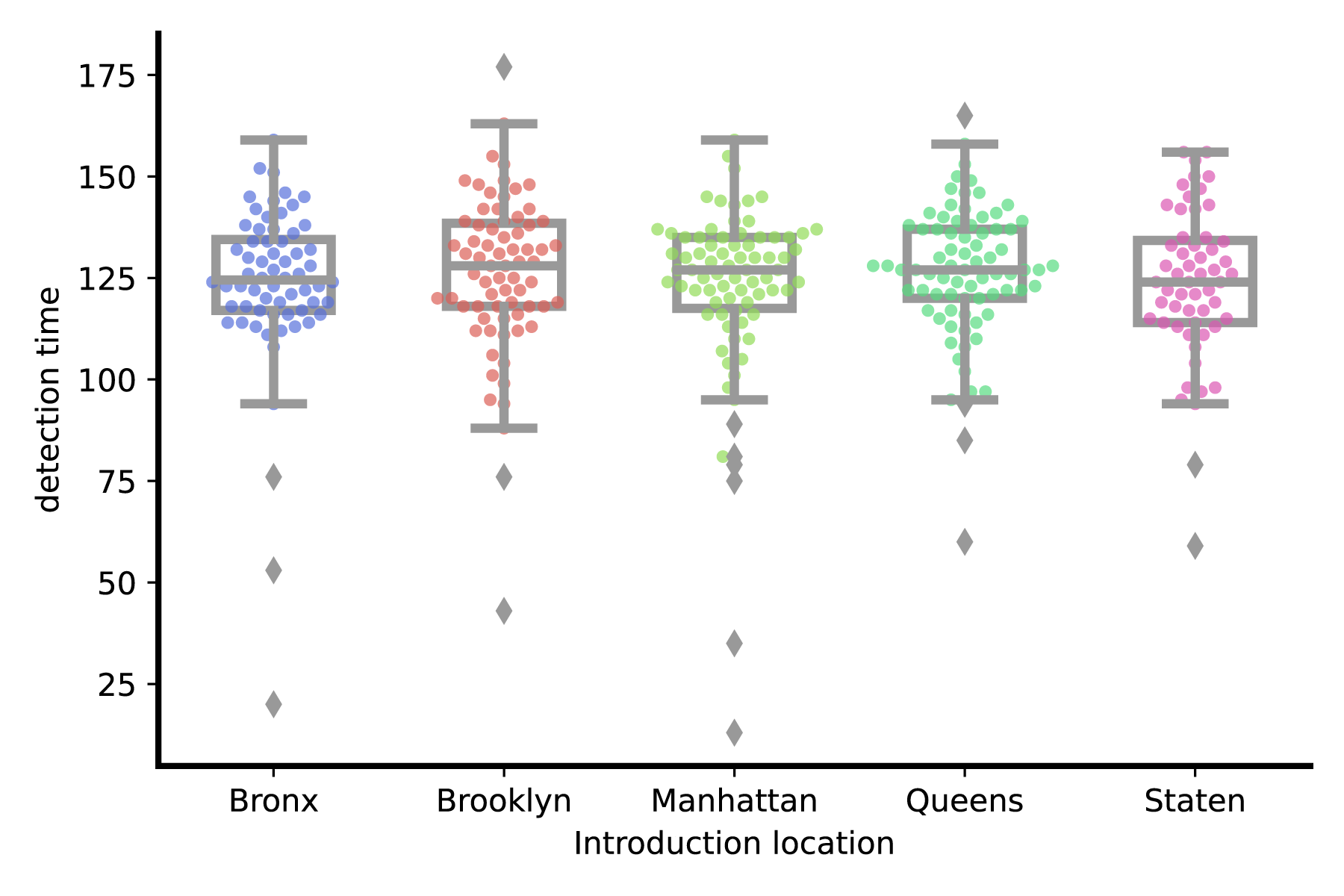

Supplement: S8 Fig — Points depict the detection time in days for each introduction location (at variant introduction 50 days after the prior variant, baseline distribution of tests, 30% of baseline test quantity, and sequencing rate 10%). Boxes and whiskers depict the minimum, lower 25%, median, upper 75%, and maximum detection times. (TIF) [file pcbi.1012416.s009.tif]
