# Supplementary material for: Surveillance strategies for the detection of new pathogen variants across epidemiological contexts
Source: PLoS Comput Biol. 2024 Sep 5;20(9):e1012416. doi: 10.1371/journal.pcbi.1012416 (PMC11407617; doi:10.1371/journal.pcbi.1012416)
Supplement: S3 Fig — Boroughs are colored by the proportion of the population that is tested each week under the baseline (A), density-based (B), and random (C) sampling strategy. Copyright: OpenStreetMap, openstreetmap.org/copyright. (DOCX) [file pcbi.1012416.s004.docx]

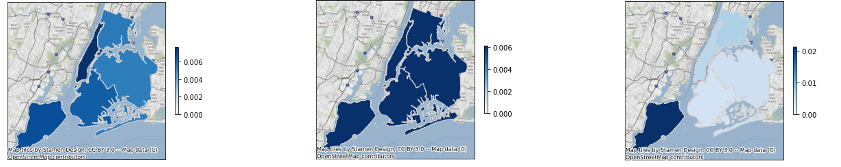


**Figure S3. Example of test rates at the borough level.** Boroughs are colored by the proportion of the population that is tested each week under the baseline (A), density-based (B), and random (C) sampling strategy.

1. Baseline B) Density C) Random
