# Supplementary material for: Surveillance strategies for the detection of new pathogen variants across epidemiological contexts
Source: PLoS Comput Biol. 2024 Sep 5;20(9):e1012416. doi: 10.1371/journal.pcbi.1012416 (PMC11407617; doi:10.1371/journal.pcbi.1012416)
Supplement: S1 Table — (DOCX) [file pcbi.1012416.s012.docx]

**Table S1. Parameters**

| **Parameter** | **Description** | **Value** | **Reference** |
| --- | --- | --- | --- |
| $p_{t}$ | *Daily test rate (per capita)* | *4.4 – 348 per 100,000 (5%-400% of baseline test level of 87 tests per 100,000)* | *NYC DPH (2)* |
| $p_{g}$ | *Daily sequencing proportion (share of positive tests)* | *0.001 – 0.9* | *NYC DPH (2)* |
| $p_{q}$ | *Control measure compliance rate* | *0.7* | *(3)* |
| $p_{TP}$ | *True positive rate of test instrument* | *0.999* |  |
| $\theta$ | *Relative transmissibility with control measure compliance* | *0.6* | *(4,5)* |
| $a_{x\{y\}}$ | *Leaky immunity parameter: relative susceptibility to infection with variant x after infection with variant y* | *0.6 (same variant), 0.5 (different variants)* | *(6)* |
| $w_{x\{y\}}$ | *Waning full immunity parameter: rate of loss of immunity against variant x after infection with variant y* | *1/90 (same variant), 1/60 (different variants)* | *(6)* |
| $L$ | *Average duration of latent period, in days* | *3* | *(7,8)* |
| $D$ | *Average duration of infectious period, in days* | *5* | *(7,9)* |
| $\mu_{contacts}$ | *Average number of close contacts, per person per day* | *4* | *(10)* |
| $b$ | *Probability of infection given a close contact with an infectious individual* | *Base variant: 0.2*  *Novel variant: 0.21-0.5* | *(11)* |
| $\epsilon$ | *Rate of ending control measures with false positive test result, per day* | *1/5* | *(12)* |
