# Supplementary material for: Surveillance strategies for the detection of new pathogen variants across epidemiological contexts
Source: PLoS Comput Biol. 2024 Sep 5;20(9):e1012416. doi: 10.1371/journal.pcbi.1012416 (PMC11407617; doi:10.1371/journal.pcbi.1012416)
Supplement: S2 Table — (DOCX) [file pcbi.1012416.s013.docx]

**Table S2. Multivariable regression results.**

| **Response variable** | **Variable** | **Coefficient** | **p-value** | **95% confidence interval** | |
| --- | --- | --- | --- | --- | --- |
| Average detection time | Test rates, multiple of baseline rates | -13.3 | 0.000 | -14.1 | -12.5 |
|  | Sequencing rate | -44.3 | 0.000 | -47.5 | -41.0 |
|  | Introduction time | 0.3 | 0.000 | 0.3 | 0.3 |
|  | Transmission probability | -603.0 | 0.000 | -627.6 | -578.5 |
|  | Random strategy | 0.5 | 0.686 | -1.8 | 2.7 |
|  | Population density-based strategy | 0.3 | 0.799 | -1.9 | 2.5 |
|  | Constant | 257.7 | 0.000 | 250.9 | 264.5 |
| Cumulative infections at detection time | Test rates, multiple of baseline rates | -124.3 | 6.3 | 0.000 | -136.9 |
|  | Sequencing rate | -502.5 | 25.9 | 0.000 | -553.7 |
|  | Introduction time | -2.1 | 0.1 | 0.000 | -2.4 |
|  | Transmission probability | 1334.0 | 193.9 | 0.000 | 952.4 |
|  | Random strategy | 6.3 | 17.5 | 0.720 | -28.1 |
|  | Population density-based strategy | 3.2 | 17.5 | 0.855 | -31.1 |
|  | Constant | 364.3 | 53.7 | 0.000 | 259.3 |
